# Digital Health Literacy About COVID-19 as a Factor Mediating the Association Between the Importance of Online Information Search and Subjective Well-Being Among University Students in Vietnam

**DOI:** 10.3389/fdgth.2021.739476

**Published:** 2021-09-27

**Authors:** Linh Hoang Thuy Nguyen, Man Thi Hue Vo, Lien Thi Mai Tran, Kevin Dadaczynski, Orkan Okan, Linda Murray, Thang Van Vo

**Affiliations:** ^1^Institute for Community Health Research, University of Medicine and Pharmacy, Hue University, Hue, Vietnam; ^2^Faculty of Public Health, University of Medicine and Pharmacy, Hue University, Hue, Vietnam; ^3^Division of Public Health, Global Health Entrepreneuship Department, Tokyo Medical and Dental University, Tokyo, Japan; ^4^Public Health Centre, Fulda University of Applied Sciences, Fulda, Germany; ^5^Centre for Applied Health Science, Leuphana University Lueneburg, Lueneburg, Germany; ^6^Interdisciplinary Centre for Health Literacy Research, Bielefeld University, Bielefeld, Germany; ^7^School of Health Sciences, College of Health, Massey University, Wellington, New Zealand

**Keywords:** COVID-19, Vietnamese students, digital health literacy, subjective well-being, mediator

## Abstract

**Introduction:** Digital health literacy (DHL) has recently been proposed as a means of enabling healthy decisions for protective behavior, preventive measures, and adherence with COVID-19 policies and recommendations especially in the era of the “infodemic”. This study aimed to (1) identify COVID-19 related DHL and its association with online information seeking; (2) to elucidate COVID-19 related DHL as a mediator predictor between the importance of online information search and its association with subjective well-being among Vietnamese university students.

**Methods:** A cross-sectional web-based survey was used to elicit the responses of Vietnamese students over 2 consecutive weeks (from April 25 to May 9, 2020, *n* = 1,003, 70.1% female students, mean age 21.4 ± 3.1). The online survey questionnaire collected data on the sociodemographic characteristics of participants, DHL about COVID-19, information seeking behavior, and subjective well-being. Mediation analysis was conducted using the importance of searching COVID-19 related information as independent variables, subjective well-being as a dependent variable, and DHL as a mediator variable.

**Results:** Among 1,003 students, the mean (SD) of DHL related to COVID-19 was 2.87 ± 0.32. In the survey, 87.2% of the students reported sufficient well-being, while almost 13% reported low or very low well-being. The findings also indicated that search engines were the most popular platform for information seeking by Vietnamese students (95.3%) and 92.8% of participants had searched for information related to the current spread of COVID-19. Not searching for hygiene regulation as part of infection control and an average level of information satisfaction were associated with limited DHL (*p* < 0.05). The importance of online information searching related to COVID-19 increased the subjective well-being of students significantly and limited DHL (*p* < 0.05). DHL was found to mediate the relationship between the importance of online information searching and the subjective well-being of students.

**Conclusion:** The finding provides insight into DHL about COVID-19 among university students, and their ability to find, understand, appraise, and use online health related information during lockdown throughout the first COVID-19 pandemic wave. DHL should be highlighted as a mediating factor that enhances the positive effect of the importance of information seeking on psychological well-being. However, further studies are needed to better define the mediating role of DHL across other factors.

## Introduction

A novel coronavirus (COVID-19) was identified on December 31, 2019, in Wuhan, China (1) and has spread to most of the countries worldwide. The WHO has identified more than 174 million confirmed COVID-19 cases, and as of June 09, 2021, 3.7 million related deaths had been reported. Among these, 224,894 cases and 3,757 deaths have been documented in Vietnam as of August 10, 2021 (2–6). In May 2021, a new COVID-19 outbreak with a new variant occurred in Vietnam, with community transmission in many provinces and cities, and lockdown measures were reinstituted throughout the country. By August 8, 2021, ~10% of the population in Vietnam had received at least one dose of a COVID-19 vaccine (4).

Vietnam has progressively implemented strict lockdown measures since the first wave of the COVID-19 pandemic in January 2020 until the recent waves, including social distancing, travel bans, screening at ports of entry, a 14-day self-isolation requirement for all international arrivals, school closures, and public event cancelations. From March 16, 2020, the wearing of masks at public venues was strictly enforced, and non-essential services were shut down nationwide at this time (7).

In response to COVID-19, the universities were closed, and face-to-face learning was pivoted to online lectures and delivered through web-based platforms and online meetings. The majority of university students have been increasingly turning to the Internet to search for health-related information, but many of them found it challenging to deal with the vast amount of COVID-19 related information (8). Digital health literacy (DHL) has been defined as “the ability to seek, find, understand, and appraise health information from electronic sources, and apply the knowledge gained for preventing, addressing, or solving a health problem” (9). Several studies before this pandemic have found that the vast majority of university students lack sufficient skills for DHL (10). For example, a moderate and low self-perceived level of DHL has been reported for nursing, medical, and health sciences university students, respectively (11–13). Up to 77% of pharmacy students were unable to evaluate the quality of the health resources they found online (14). Thus, it can be assumed that searching for COVID-19 related information through the Internet and making health decisions is challenging for many of them (15).

The quality of online information available has played a crucial role in health promotion during the pandemic. However, this has occurred in a context of an overabundance of true, false, and mixed information, including on COVID-19 diagnosis, treatment, protective behavior, preventive measures, dashboard statistics, and public health recommendations. This phenomenon has been entitled the “infodemic,” which spreads faster than the pandemic itself, especially through online social media platforms (16). This “information overload” may lead to feelings of confusion and stress (17). Hence, a sufficient ability to search for and appraise health-related information can be expected to have positive effects on the health behaviors in preventing diseases and mental health outcomes, such as well-being (9).

The health-related consequences were manifold and the pandemic, in particular, caused mental health problems and health communications problems with adverse effects on the mental health of the population. A recent study in the United States reported that 31% of adults had symptoms of anxiety or depression during the pandemic (18). These surging issues are related to the spread of myths and misinformation about this epidemic and associated fear and stress (7, 9, 10).

COVID-19 control measures such as social distancing measures, school closure or decreased physical activities, fewer locations visited, and increased smartphone use seemed to have an immediate negative affect on the emotional well-being of university students, characterized by increased anxiety and depression (19, 20). Furthermore, there were many social factors associated with the subjective well-being of university students that were affected by the pandemic, including family income, which may have become more unstable due to job loss during the pandemic, having a relative or acquaintance infected with COVID-19, and delays in academic progress (20). Health literacy has been identified as one factor associated with subjective well-being among adults (21). However, evidence about the role of DHL as a mediating factor between the importance of online information search and mental health among students is scarce in the context of the COVID-19 pandemic.

Most of the studies published to date have separately analyzed the predictors and outcomes of health literacy, while only a few have evaluated the possible role of health literacy as an intermediate pathway that affects the relationship between potential determinants and well-being (22). To date, it is unknown whether health literacy mediates the association between the quality of information searched on the Internet and subjective well-being and this is the main reason why we might explore to what extent DHL mediates the association between the importance of online information as perceived and subjective well-being during the COVID-19 pandemic in Vietnam.

This study aimed (1) to investigate COVID-19 related DHL and its association with online information seeking; (2) to elucidate if COVID-19 related DHL plays a mediating role in the relationship between perceptions about the importance of online information searching and subjective well-being among Vietnamese university students.

## Methods

### Study Design

This was an online cross-sectional survey. Participation was voluntary, with participants answering once and during a 2-week period (from April 25 to May 9, 2020).

### Study Procedures

This research is part of a large-scale COVID-Health Literacy (HL) research network that was launched in mid-March in 2020 and now includes around 50 countries worldwide (23). Data collection in Vietnam was conducted through an online survey hosted by a secured website (Microsoft Forms online). The COVID-HL questionnaire [23] was adapted to the local Vietnamese context. The process of adaptation included a translation from English into Vietnamese, a pilot test with 10 volunteers, and refinement based on the results of the pretest before official use. The whole questionnaire took approximately 10–15 min to complete. The recruitment was the official dissemination of the survey invitation to Vietnamese university students *via* various social media platforms. All participants took part in the survey on a voluntary basis, could withdraw without justification at any point, and their confidentiality and anonymity were fully protected. The online questionnaire was kept open for 2 weeks only. The study participation was voluntary and before accessing the questionnaire, the participants provided consent. The study was approved by the Ethical Review Committee of Hue University of Medicine and Pharmacy, Vietnam (No. H2020/050 dated April 20, 2020).

### Sample Size and Sampling

For this study, a convenience sample with a snowball approach was used: the more university students that completed the online questionnaire, the more widespread it became, as participants were asked to share the survey web link with their friends and relatives. A link to the survey was included in the invitation of the study objective and participation.

Data were analyzed for respondents who met the following inclusion criteria: (1) being at least 18 years old, (2) Vietnamese citizens who understood the Vietnamese language and resided in Vietnam at the time of the study, and (3) being a university student and searching for COVID-19 related information during the first wave of the COVID-19 pandemic in Vietnam. The final sample for analysis included 1,003 university students from 1,055 responses (52 participants who did not meet the inclusion criteria listed above were excluded). Most of the participants were studying at Medical or Health Science Universities (83.1%), 70.1% were female students, and the mean (SD) age of this sample was 21.4 (±3.10).

### Variables

The variables include sociodemographic information, such as gender (male/female/diverse), age (continuous in absolute numbers), study program (bachelor/masters/other), anxiety about the future, personal health status, online information seeking behavior, and the importance of searching for information. All of these variables and their measurements were well-described in the survey guidelines of the international COVID-HL network (https://covid-hl.eu/) (24).

### Online Information Seeking Behavior

This included different online sources for information searching, different topics that were searched for with regard to COVID-19, the language of the information searched, and satisfaction with the information found.

A list of 10 online sources for searching information on COVID-19 and related topics were used e.g., search engines (Google and Yahoo), websites of public institutions, Wikipedia, and social media platforms (Facebook, Instagram, and Twitter). These items could be answered on a four-point scale ranging from 1 (don't know) to 4 (often). These variables were recoded and classified as “yes,” which included “often/sometimes,” and “no,” which included “rarely,” “never,” and “don't know.”

Regarding COVID-19 related topics, a list of nine topics was provided, with a possible response of “yes” or “no.” This list included, e.g., the current spread of COVID-19, transmission routes, symptoms and protective measures, assessment the of current situation, restrictions, economic and social consequences, and dealing with psychological stress.

Participants were also asked about their level of satisfaction with the information they found on the Internet about COVID-19 and related topics. Questions were answered on a five-point Likert scale ranging from 1 (very dissatisfied) to 5 (very satisfied).

#### The Importance of Searching Information Related to COVID-19

The importance of searching information related to COVID-19 was measured using six items which were introduced with “How important is it to you that…?” and included six dimensions of information importance: (1) being updated; (2) verified; (3) quickly learning the most important things, (1) information from official sources, (5) represented different opinion, and (6) dealt with comprehensively. All items could be rated on a four-point Likert scale ranging from 1 (not important at all) to 4 (very important).

#### Digital Health Literacy

Digital health literacy was measured using an adapted and translated version of the Digital Health Literacy Instrument (DHLI). The DHLI tool is an instrument that proved to show as acceptable to good reliability in several previous studies (25–27). For the context of this study, all items were adapted to the specific context of COVID-19, and previous studies from the COVID-HL network could show this adapted tool to be valid and reliable for capturing DHL in university students during the pandemic.

For the context of this study, the items were adapted to information about COVID-19. The original instrument included 21-items assessing seven DHL skill dimensions: (1) information searching, (2) adding self-generated content, (3) evaluating reliability, (4) determining relevance, (5) protecting privacy, (6) operation skills, and (7) navigation skills. For this study, we included five skills (1–5) with each dimension containing three items which could be answered on a four-point response scale, ranging from 1 (very difficult) to 4 (very easy), with higher scores representing higher DHL (e.g., when you search the Internet for information on COVID-19 or related topics, how ease or difficult is it for you to …) or from “never” to “often” for questions related to protecting privacy (e.g., when you post a message about the COVID-19 or related topics on a public forum or social media, how often…). The internal consistency of the overall DHLI score was acceptable (Cronbach's alpha = 0.77) and the subscales reached acceptable to excellent reliability scores (0.71 < Alpha< 0.95). After recoding all items (with higher values indicating a higher DHL), the mean values for each subscale and the DHLI scale were created. With regards to the statistical analyses, we dichotomized the general DHL using a median split (0 = sufficient DHL, 1 = limited DHL).

#### The Subjective Well-Being of University Students

The subjective well-being of university students as a dependent variable was measured using the WHO-5 well-being index (28). This instrument included five items that could be answered on a 6-point Likert scale ranging from 0 (“at no time”) to 5 (“all of the time”). According to the scale developer, the raw score for each item has been multiplied by 4, resulting in a transformed scale from 0 (lowest well-being) to 100 (highest well-being). The subjective well-being scale reached excellent reliability (Alpha = 0.91). The existing cut-off points suggest a low well-being for score ≤ 50, while values ≤ 28 indicate symptoms of depression (very low well-being) (29).

### Data Analysis

**Figure 1:**
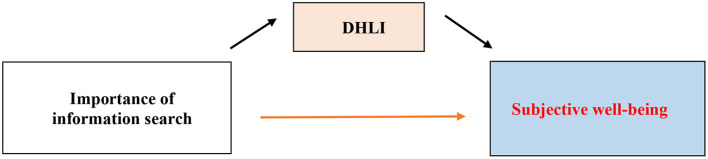
The conceptual framework of study.

The descriptive statistics were presented as mean ± SD for continuous data, while the categorical variables were presented as percentages. The associations between the independent variables and the dependent variable “limited DHL” were investigated with bivariate analysis (chi-squares and *t*-tests). Regarding the effect size, we calculated the Cramér's V (for chi-square test) or Cohen's *d* (for *t*-tests) (30). For Cramér's V, the effect was small at ≥0.1, medium at ≥0.3, and large at ≥0.5. For Cohen's *d*, the following effect was ≥0.2 (small), ≥0.5 (medium), and large at ≥0.5 (30). With the statistically significant differences in this study, the value of Cramér's V and Cohen's *d* were more than 0.1 and 0.2, respectively. A multivariable logistic regression model was used to analyze the association between the limited levels of DHL and associated factors after adjusting for sex and age. This form of regression analysis was chosen because there are no predefined cut-off values for the dependent variable resulting in an empirical division of low vs. high DHL (using a median split). In the next step, the mediation analyses were conducted to test the direct effects of the predictor as an independent variable (importance of information search), on well-being as the dependent variable, and the indirect effect through the mediator (DHL). According to the mediation model as discussed by Baron and Kenny (31), we explored DHL as a mediator of the association between the independent and the dependent variable (as shown in Figure 1).

The mediation model was tested separately following the procedure outlined by Haye et al. (32), using the modeling tool PROCESS (version 3.5) for SPSS. A Bootstrap approach (using 1,000 resamples) was utilized to assess the statistical significance of the mediation by means of the confidence interval around the “indirect effect estimate.” Bootstrapping is a robust non-parametric technique for hypothesis testing and effect size estimation without making the assumptions of normal distribution.

The statistical analyses were performed using SPSS version 20.0 (IBM Corp., Armonk, NY, USA). In all the analyses, *p* < 0.05 was regarded as indicating statistical significance.

## Results

Table 1 shows that mean of DHL was 2.87 ± 0.32. The students reported most difficulties in the DHLI dimension “*adding self-generated content”* had the lowest mean (2.25 ± 0.88), while for *protecting privacy*, the highest mean values could be observed (3.41 ± 0.71). As for subjective well-being, 87.2% of the participants showed sufficient well-being (50 scores), while almost 13% reported low or very low well-being.

**Table 1:** Digital health literacy (DHL) related to COVID-19 and the subjective well-being (*n* = 1,003).

	*n* ^*^	M (SD)^*^	%^*^
**Digital health literacy**			
General digital health literacy (DHL)		2.87 (0.323)	
**Subscale**			
Information searching		3.06 (0.438)	
Adding self-generated content		2.25 (0.877)	
Evaluating reliability		2.73 (0.492)	
Determining relevance		2.89 (0.404)	
Protecting privacy		3.41 (0.709)	
Subjective well-being		71.44 (±18.92)	
Sufficient well-being	873		87.2
Low well-being	92		9.2
Very low well-being	36		3.6

* *M, mean value; SD; n, frequency; %, percent*.

Table 2 shows that search engines were the most popular sources used for online information seeking on COVID-19 related topics (95.3%), followed by social media (92.4%) and news portals (91.6%). With regard to the topics, 92.8% of participants searched for information related to the current spread of the coronavirus, while 37.7% of participants searched for information about dealing with psychological stress caused by COVID-19. Guidebook communities (e.g., poster, brochure, and guidelines in communities), health portals (e.g., ministry of health website) in sources of information seeking, “hygiene regulations” among searching topic and information satisfaction showed significant differences according to DHL level (*p* < 0.05). In addition, the mean score of the importance of seeking information related to COVID-19 differed significantly by DHL level (*P* < 0.05), i.e., the respondents with limited DHL also attached lower importance to information search.

**Table 2:** COVID-19 related information-seeking behavior and attitude toward COVID-19 related online information searching among Vietnamese university students by DHL.

**Variables**	**General digital health literacy (DHL)**						***P***
	**Sufficient**		**Limited**		**Total**		
	***N***	**%**	***n***	**%**	***N***	**%**	
**Sources used for online COVID-19 information seeking (often/sometimes)**							
Search engines	476	49.8	480	50.2	956	95.3	0.649
Websites of public bodies	388	51.2	370	48.8	578	57.6	0.168
Wikipedia and other online-encyclopedias	322	49.7	326	50.3	648	64.6	0.825
Social media	462	49.8	465	50.2	929	92.6	0.804
YouTube	390	50.3	386	49.7	776	77.4	0.719
Blogs on health topics	267	50.1	266	49.9	533	53.1	0.923
Guidebook-communities	268	53.5	233	46.5	501	50.0	0.025
Health portals	376	52.7	337	47.3	713	71.1	0.006
Websites of doctors or health insurance companies	282	51.6	264	48.4	546	54.4	0.24
News portals	467	50.8	452	49.2	919	91.6	0.07
**Topics searched for in the context of the COVID-19 (yes)**							
Current spread of the COVID-19	478	50.3	473	49.7	951	94.8	0.397
Transmission routes of the COVID-19	351	50.1	342	49.4	693	69.1	0.508
Symptoms of the disease COVID-19	414	50.5	406	49.5	820	92.8	0.471
Individual measures to protect against infection	417	50.8	404	49.2	821	81.9	0.258
Hygiene regulations	320	52.5	290	47.5	610	60.8	0.048
Current situation assessments and recommendations	320	51.5	301	48.5	621	61.9	0.202
Restrictions	324	50.5	317	49.5	641	63.9	0.616
Economic and social consequences of the COVID-19	289	51.2	276	48.8	565	56.3	0.388
Dealing with psychological stress caused by the COVID-19	200	52.9	178	47.1	378	37.7	0.145
**Information satisfaction**							
Dissatisfied	33	52.4	30	47.6	63	6.3	<0.001
Average	180	41.1	258	58.9	438	43.7	
Satisfied	288	57.4	214	42.6	502	50.0	
	**Mean**	**SD**	**Mean**	**SD**	**Mean**	**SD**	
Importance of searching information related to COVID-19	3.66	0.316	3.61	0.33	3.64	0.333	0.023^*^

* *t-test for compare means*.

In particular, in Table 3, the participants who did not search for information on “infection control” had an increased odds ratio (*OR*) of having a limited DHL compared with those respondents who searched for information on this topic (*OR* 1.35, 95% *CI* 1.04–1.74). As for the relationship between information satisfaction and DHL, the students with average information satisfaction had an increased *OR* of having a limited DHL (*OR* 1.90, 95% *CI* 1.46–2.47).

**Table 3:** Association between DHL and information seeking behavior among Vietnamese university students.

		**Limited DHL**			***P***
		AOR^*^	**95% CI**		
**Sources used for online COVID-19 information seeking**					
Guidebook-communities					
	No	1.000			
	Yes	0.807	0.622	1.047	0.106
Health portals					
	No	1.000			
	Yes	0.722	0.543	0.961	0.025
**Topics searched for in the context of the COVID-19**					
Hygiene regulations					
	Yes	1.000			
	No	1.347	1.041	1.743	0.023
**Information satisfaction**					
	Dissatisfied	1.325	0.778	2.255	0.301
	Average	1.898	1.459	2.468	<0.001
	Satisfied	1.000			
**Importance of searching information**		0.635	0.431	0.936	0.022

* *AOR, adjusted odds ratio (adjusted for gender,age)*.

**Figure 2:**
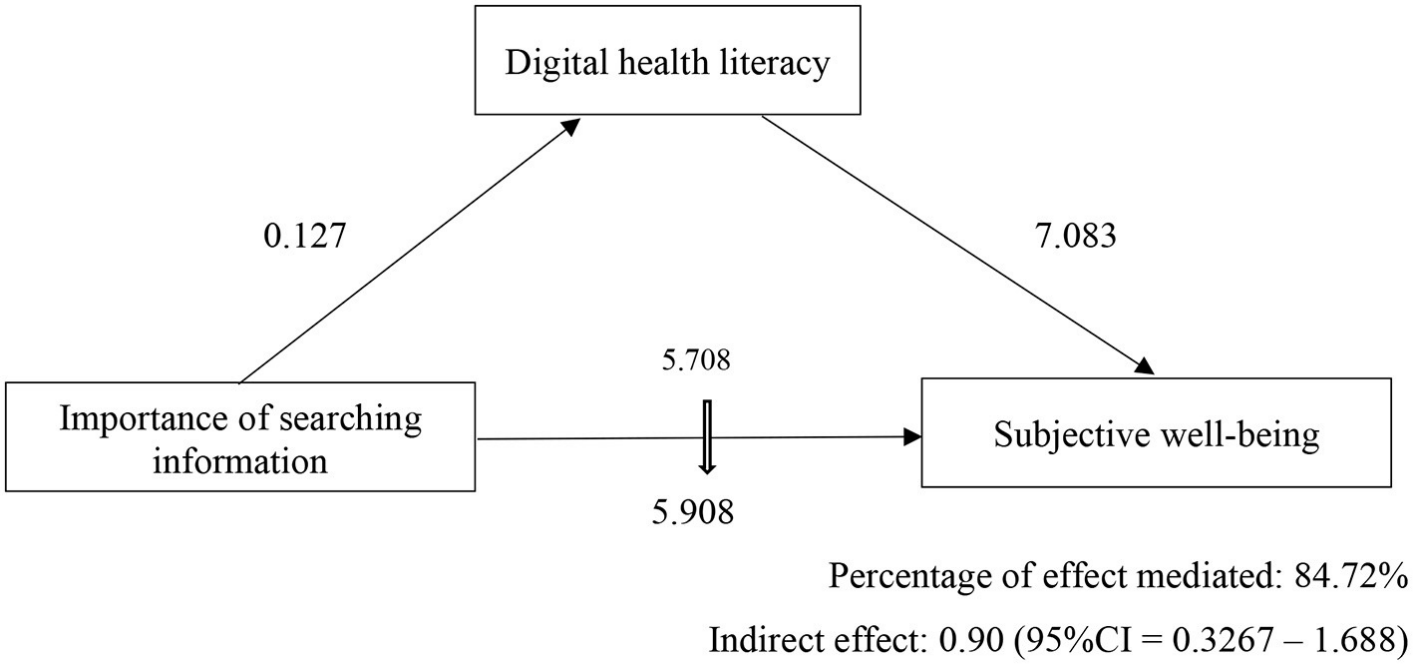
Digital health literacy mediates the relationship between the importance of searching information and subjective well-being (*n* = 1,003).

The mediation analyses suggest that DHL partially mediated the relationship between the importance of searching for information and subjective well-being. The “indirect effect” of the importance of searching for information on psychological well-being through the DHL mediator was 0.90 (95% *CI* = 0.3267–1.688). The direct effect on subjective well-being remained significant [5.91 (95% *CI* = 2.321–9.495)] and was increased with the mediator input (DHL) to 6.81 (95% *CI* 3.229–10.392) (as shown in Table 4; Figure 2).

**Table 4:** Digital health literacy as a mediator of the relationship between the importance of searching information and subjective well-being.

**Mediator model**	**Beta (95% CI)**			***P***
**General DHL as dependent variable**				
Importance of searching information	0.127	0.066	0.189	<0.001
**Outcome model**				
General DHL	7.083	3.473	10.692	0.0001
Importance of searching information	5.708	2.321	9.495	0.0013
	B	95% CI		*P*
Total effect	6.811	3.229	10.392	0.0002
Direct effect	5.908	2.321	9.495	0.0013
Indirect effect	0.903	0.3267	1.6881	

## Discussion

Our study findings indicated that Vietnamese university students have a considerably high overall DHL score (2.87 ± 0.323), which is comparable with the results of a previous report among the university students of Pakistan (33). Search engines were utilized as the most popular platform for information seeking, followed by social media. The majority of the participants looked for information about the current spread of COVID-19. A lack of content for “infection control” during information searching, in particular, not searching for hygiene regulation, and an average level of information satisfaction were associated with limited DHL. Interestingly, DHL was found to mediate the relationship between the perceived importance of searching for information and their subjective well-being.

Search engines and social media were the most popular sources for health information seeking for Vietnamese university students. According to the Global Digital suite of reports in 2018, 64 million out of 96 million Vietnamese people were online, and 89.1% of online users are active social media users (34). Besides that, during the COVID-19 breakout, the Vietnamese Ministry of Health has utilized social medial channels as an additional way to share well-timed information about COVID-19 to the population (35). Therefore, the government can make use of social media by integrating the official health news into social media platforms popular in Vietnam (Facebook, Youtube, etc.) in future outbreaks in a timely manner.

Regarding the effect size which measures the strength of the relationship between the two variables, in all the statistically significant differences, the value of Cramér's V and Cohen's *d* were more than 0.1 and 0.2, respectively. The results of this effect size were acceptable to determine the difference is real (30). Research design, sample size, type of measurement, etc., could influence the effect sizes (36). Therefore the magnitude of effect size, which was detected to be significant in our study, should be considered in relation to the research design. In this regard, our effect size showed a rather positive association between searching for online information and DHL, next action research (community intervention or randomized controlled trial) in which online information-based intervention is expected to improve better subjective well-being through increased DHL is quite feasible.

As there has been widely shared disinformation on the social platforms during the pandemic, DHL of university students is affected when students cannot properly appraise the quality of information on COVID-19 and are manipulated by false information. The overabundance of COVID-19 related information could bring about negative mental health consequences to those who are not able to critically evaluate the information they read (37), as disinformation and fake news could lead to confusion and increased stress (38, 39). As the Internet and digital platforms have become the major sources of health information regarding COVID-19, it is important to strengthen the DHL of students. However, there is a clear lack of interventions that strengthen DHL for young adults so far.

The importance of information content while searching, as well as information satisfaction, was presented by their relationship with general DHL. The students with average information satisfaction were more likely to have limited DHL compared with those who were satisfied with the information obtained. This might be because the higher levels of DHL require one to critically choose and evaluate available information on the Internet (40). DHL is also considered to involve various different types of literacy, such as health literacy and information literacy (41) that may enhance the confidence of participants in accessing the information and in turn, their satisfaction. This finding also corresponds with a previous study that health literacy could be a contributing factor to information satisfaction (42) when lower health literacy is associated with less perceived information provision and less satisfaction with the information.

However, the students who were dissatisfied with the information obtained also had a higher adjusted odds ratio (AOR) for a limited DHL, but this association was not statistically significant in our data analysis. It may also be that low satisfaction can be attributed to respondents being more critical of available information, indicating a higher DHL. This might be especially true for people with chronic diseases who reported lower satisfaction with COVID-19 information in a Chinese study (43). Therefore, it will need further investigation to clarify this finding. Similar findings were also shown in the recent studies in Germany and Portugal, on the association between DHL and information found online and on social media (8).

Despite the fact that the prevalence of low and very low subjective well-being among our study participants was found to be lower than the findings of a study among preclinical medical Vietnamese students (44), our model revealed the mediating role of DHL in the relationship between the perceived importance of COVID-19 related information search of students and their subjective well-being. In particular, DHL could constitute an important pathway by which the importance of information searching on COVID-19 improves the subjective well-being of students. Regarding the association between online seeking behavior, DHL, and well-being, the results of our study are in line with the scientific pre-pandemic evidence (45, 46). In particular, the effect of the importance of information searching on COVID-19 on subjective well-being was enhanced with DHL. As information seeking was seen as purposeful and driven by personal need (47), it is possible that the individuals who perceive searching for COVID-19-related topics as important are more likely to actively seek this information than the individuals who do not. Consequently, this provides them richer knowledge of online health information and in turn, positively affects well-being through improving the health knowledge and self-care skills (48, 49). Furthermore, health literacy, as a basic component of DHL, is associated with frequent online information search (50), and also with better health behaviors (51), which in turn enhance the well-being of an individual (52), which can be the explanation for the indirect effect of the importance of COVID-19 information searching on subjective well-being through DHL during the pandemic in our study.

Considering the tightening of the pandemic measure due to the serious re-emergence of COVID-19 in Vietnam since late April 2021 [47], the findings from this study are meaningful for informing future research, where DHL is included along with other established mediators that link other factors to well-being, such as mental, physical, and social health–the commonly known combination of well-being in a time of virtual information during the pandemic. Apart from deepening the research concerning these underlying mechanisms, the mediating role of DHL may have important implications for health promotion interventions, as DHL can be improved more easily with the change in positive perception about the importance of COVID-19 related information search. Consequently, in the digital era, it is well-noted that online health resources should be designed in a way that fits the literacy levels of the target users. This is especially crucial during pandemics when online health information does not only provide users with useful information but also can help to enhance the health and well-being of individuals.

Our study has some limitations that should be acknowledged. First, our data cannot be considered representative of all university students in Vietnam and the results cannot be generalized due to the use of convenience sampling. The more university students completed the online questionnaire, the more widespread it became, as participants were asked to share the survey web link to their friends and relatives. The recruitment was used and went out from our medical university; therefore, it could be that medical and health science students with their dominant samples (83.1%) have a higher DHL level as they have more background information and scientific expertise. Second, the cross-sectional design does not allow us to interpret causation but is a useful first step for developing a research hypothesis. As the survey used self-reported data, recall bias could have occurred. Last, as social media were used as part of the recruitment strategy, this could have led to the selection bias excluding the number of students without access to digital media during the COVID-19 pandemic. We also expect that the DHLI-Covid tool has proven to be valid and reliable and that a Vietnamese validation study is on its way and will be hopefully published soon (48, 49).

## Conclusions

This study has provided insightful evidence about the DHL of Vietnamese students, and their ability to properly find, understand, appraise, and use online health related information during the COVID-19 epidemic. Moreover, the results suggest that DHL during the pandemic may serve as a pathway by which attitudes toward information searching bring a positive effect on subjective well-being in the digital era. However, as there is limited research on the topic, future studies that take into account the possible effect of DGL, other potential moderators, and specific health outcome measures are needed to define the role of DHL in mediating model in a more comprehensive way. The efforts of governments to improve health literacy based on the digital platform can significantly contribute not only to COVID-19 prevention and control but also increase the well-being of students.

## Data Availability Statement

The raw data supporting the conclusions of this article will be made available by the authors, without undue reservation.

## Ethics Statement

The study was conducted according to the guidelines of the Declaration of Helsinki and approved by the Ethical Review Committee of the University of Medicine and Pharmacy, Hue University, Vietnam (No H2020/050 dated 20/04/2020). Anonymity and informed consent were ensured *via* online registration in our survey during the COVID-19 pandemic.

## Author Contributions

TV, LN, OO, and KD contributed to the study design and conceptualization. LN, LT, MV, and TV did the statistical analysis, interpretation, data, and drafting of the initial manuscript. LN, LT, and TV coordinated the questionnaire adaptation and data collection. TV, LN, LT, MV, KD, OO, and LM critically revised the draft manuscript. All authors have read and approved the final manuscript.

## Funding

This study received financial support from the Institute for Community Health Research, University of Medicine and Pharmacy, Hue University, Vietnam.

## Conflict of Interest

The authors declare that the research was conducted in the absence of any commercial or financial relationships that could be construed as a potential conflict of interest.

## Publisher's Note

All claims expressed in this article are solely those of the authors and do not necessarily represent those of their affiliated organizations, or those of the publisher, the editors and the reviewers. Any product that may be evaluated in this article, or claim that may be made by its manufacturer, is not guaranteed or endorsed by the publisher.
